# A Fracture Analysis of Ti-10Mo-8V-1Fe-3.5Al Alloy Screws during Assembly

**DOI:** 10.3390/ma9100852

**Published:** 2016-10-19

**Authors:** Weifang Zhang, Yuanxing Huang, Wei Dai, Xiaoshuai Jin, Chang Yin

**Affiliations:** 1 Science & Technology on Reliability & Environment Engineering Laboratory, Beihang University, Beijing 100191, China; zhangweifang@buaa.edu.cn (W.Z.); linhaihyx@126.com (Y.H.); jinxiaoshuai@buaa.edu.cn (X.J.); frank_yinchang@163.com (C.Y.); 2 Beijing Institute of Aeronautical Materials, Beijing 100095, China

**Keywords:** titanium alloy, screw, brittle fracture, internal defect, grain boundary cracking

## Abstract

Titanium screws have properties that make them ideal for applications that require both a high strength-to-weight ratio and corrosion resistance, such as fastener applications for aviation and aerospace. The fracture behavior of Ti-10Mo-8V-1Fe-3.5Al (TB3) alloy screws during assembly was explored. Besides visual examination, other experimental techniques used for the investigation are as follows: (1) fracture characteristics and damage morphology via scanning electron microscopy (SEM); (2) chemical constituents via energy dispersive spectroscopy (EDS) and hydrogen concentration testing; (3) metallographic observation; (4) stress durability embrittlement testing; and (5) torsion simulation testing. Results show that the fracture mode of the screws is brittle. There is no obvious relation to hydrogen-induced brittle. The main reason for the fracture of titanium alloy screws is internal defects, around which oxygen content is high, increasing brittleness. The internal defects of screws result from grain boundary cracking caused by hot forging.

## 1. Introduction

Titanium alloys are widely used to fabricate aviation fasteners due to their high strength, good thermal stability, and excellent corrosion resistance [1]. Two kinds of materials are mainly used in titanium alloy fasteners: alpha-beta alloys such as Ti-6Al-4V (TC4) and Ti–3Al–4.5V–5Mo (BT16), and beta alloys such as Ti-10Mo-8V-1Fe-3.5Al (TB3). TC4 titanium alloys exhibit low ductility at ambient temperatures [2,3]. Therefore, fasteners with TC4 are usually formed via hot-heading, and vacuum solid solutions and aging treatment are conducted, for which the production cost is high [4,5]. One characteristic of BT16 titanium alloys is good deformability at ambient temperatures such that fasteners with BT16 can be produced via cold-heading, while the strength needs to be guaranteed via cold deformation strengthening [6,7]. TB3 titanium alloys, which usually exhibits relatively low strength but high ductility, are often treated via cold-heading and consequently aged to high strength, which could reduce costs [8].

Brittle fracture is one of the most serious failure modes of titanium alloy fasteners that can be applied to the key structures. Once the brittle fracture of titanium alloys occurs, it can cause catastrophic accidents. However, the brittle fracture mechanism of titanium alloys is very complicated, which makes the process of root causes failure analysis very difficult. For example, oxygen embrittlement, liquid metal embrittlement, hydrogen embrittlement, room temperature creep, stress corrosions, and internal defects as well as the above coupling mechanisms can lead to the brittle fracture of titanium alloys. The contribution of manufacturing process of different fasteners to the above failure mechanism is different. For example, oxygen and hydrogen occur in the heat treatment process [9,10]; internal defects such as the inclusion and brittle segregation are induced during the melting process [11]; liquid metal embrittlement agents are introduced during welding process [12]; titanium alloys exhibit room temperature creep when subjected to stresses close to yield stress for an extended period of time [13,14,15,16]. The screw failures during assembly involve the availability of the same batch screws and relates to the requirement for disassembly of the assembled screws. As a result, it is of great significance to find the main failure mechanism and the corresponding manufacturing process that could lead to brittle fracture.

Twenty-five thousand screws were produced in the same batch. Two hundred TB3 alloy screws were assembled, and four were fractured. In this paper, the fracture behavior of titanium screws during assembly was explored. Herein, the main failure mechanism of fracture, which was ascertained to be brittle fracture by visual inspection and microscopic observation, together with the screws’ manufacturing process leading to such failure, is investigated.

## 2. Experimental Procedure and Results

The geometric specifications of this TB3 alloy screws are as follows: the internal diameter is 3.4 mm, the screw pitch is 0.7 mm, and the length is 14 mm. The manufacturing process of the titanium wire includes vacuum melting, hot forging, rolling, cold drawing, and dehydrogenation. The manufacturing process of the screw includes titanium wire, cold heading, punching square hole, thread rolling, dehydrogenation annealing, fluorescent penetrant inspection, coating MoS_2_—which serves the purpose of solid lubricant—and packing. The screws were applied loads via torque wrench. The maximum torque was set to 2.5 N·m, and the loading sequence was clockwise. The four fractured screws were numbered #1–4.

### 2.1. Visual Inspection

**Figure 1:**
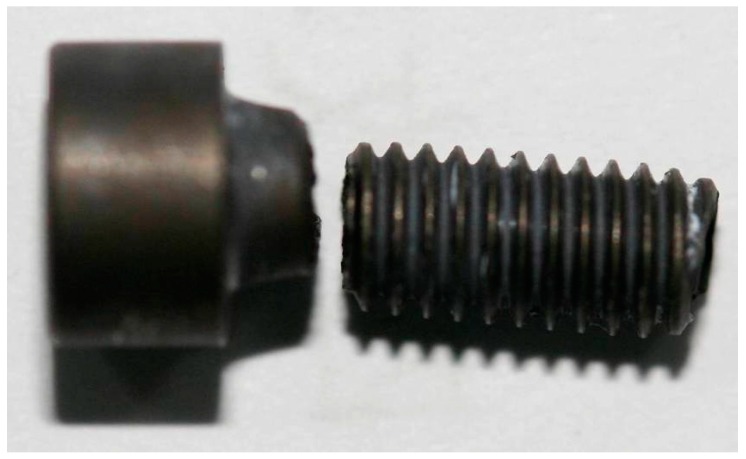
Fracture position of screw.

**Figure 2:**
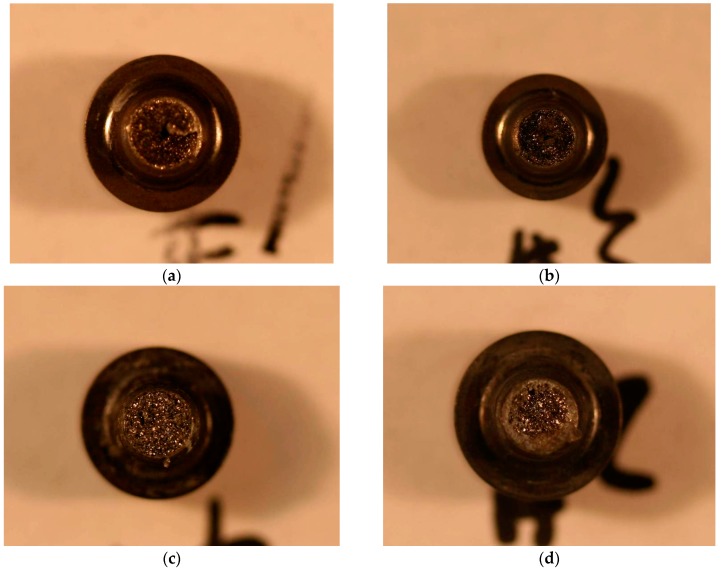
Fracture surface macro appearance of the (**a**) #1; (**b**) #2; (**c**) #3; and (**d**) #4 screws.

The positions of the fracture are all at the root of the screw thread, as shown in Figure 1. As shown in Figure 2a,b, the macroscopic fracture characteristics of the #1 and #2 screws are substantially the same: (1) the center of the screw fracture appears dark-colored, and some axial cracks on it are similar to void; (2) a large number of reflective facets (reflective facet) are shown, and there is no reflective facet around the fracture edge, which appears gray; (3) no macroscopic plastic deformation was visible. As shown in Figure 2c,d: (1) The color of the #3 and #4 screws fracture centers are brighter compared with the #1 and #2 fractures, and there are also a large number of reflective facets as well as many non-reflective facets around the gray fracture edge; (2) the gray area of the #3 fracture edge is relatively larger; (3) no obvious plastic deformation exists.

### 2.2. Microscopic Observation

**Figure 3:**
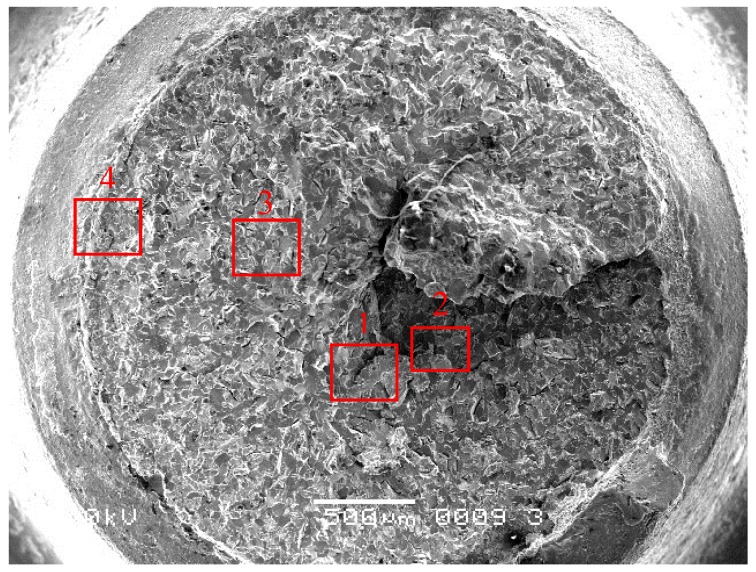
Low magnification of fracture surface of #1 screws (500 μm).

**Figure 4:**
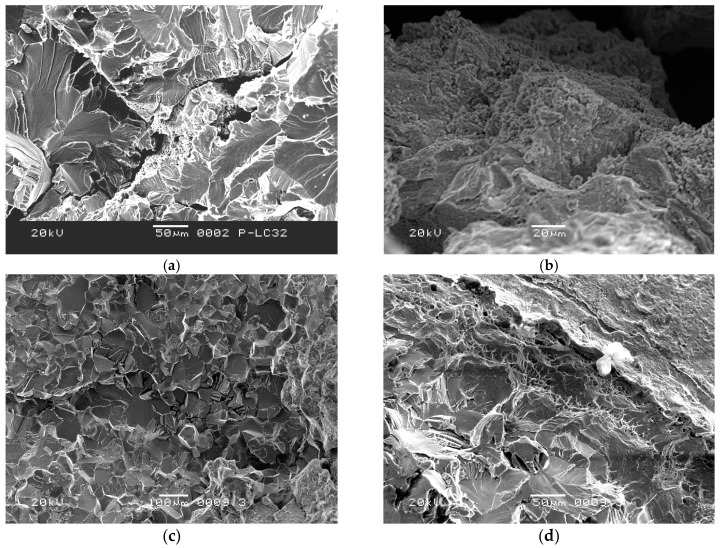
(**a**) Axial cracking and cleavage characteristics in the center of the fracture surface (Region 1 in Figure 3, 50 μm); (**b**) High magnification of the axial crack (Region 2 in Figure 3, 20 μm); (**c**) Grain boundary cracking characteristics around the axial crack (Region 3 in Figure 3, 100 μm), (**d**) Dimple morphology in margin of the fracture surface (Region 4 in Figure 3, 50 μm).

The four fractured screw specimens were firstly dipped into an acetone solution and cleaned with an ultrasonic washing machine for 5 min. Then, they were observed with an FEI Quanta 600 Field Emission scanning electron microscope (SEM). The #1 and #2 screws have the same microscopic appearance. Figure 3 shows the entire fracture surface of #1 screw. The axial crack can be clearly seen in the center of the fracture surface. Grain boundary cracking characteristics were observed around the axial crack. The major fracture surface morphology of the screw around the axial crack is a cleavage river pattern, occupying the entire fraction of the fracture surface, as shown in Figure 4a (Region 1 in Figure 3). Judging from the river pattern, the axial crack is the fracture origin. A high magnification of the axial crack indicates that the crack defects were formed during the manufacturing process, as shown in Figure 4b (Region 2 in Figure 3). Small granular white objects are visible in Figure 4b. The grain boundary cracking characteristics around the axial crack can be seen in Figure 4c (Region 3 in Figure 3). Morphologies such as dimple can be seen in the margin of the fracture surface, as shown in Figure 4d (Region 4 in Figure 3).

**Figure 5:**
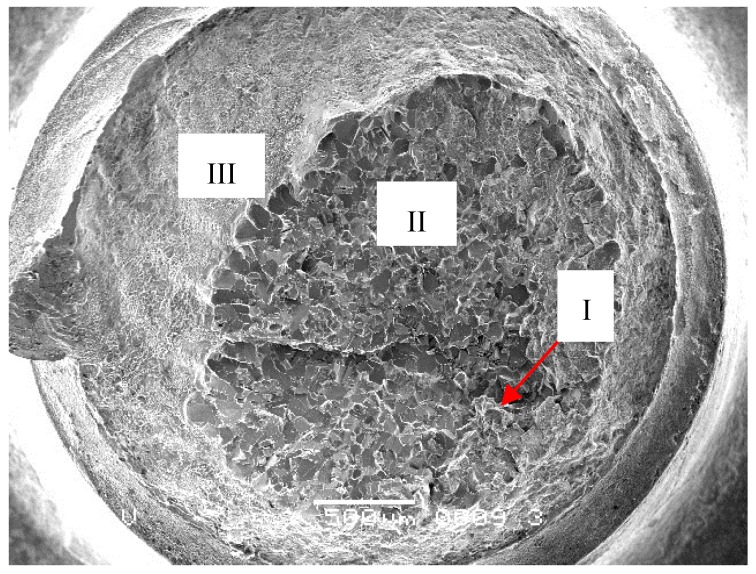
Low magnification of fracture surface of the #4 screw (500 μm).

**Figure 6:**
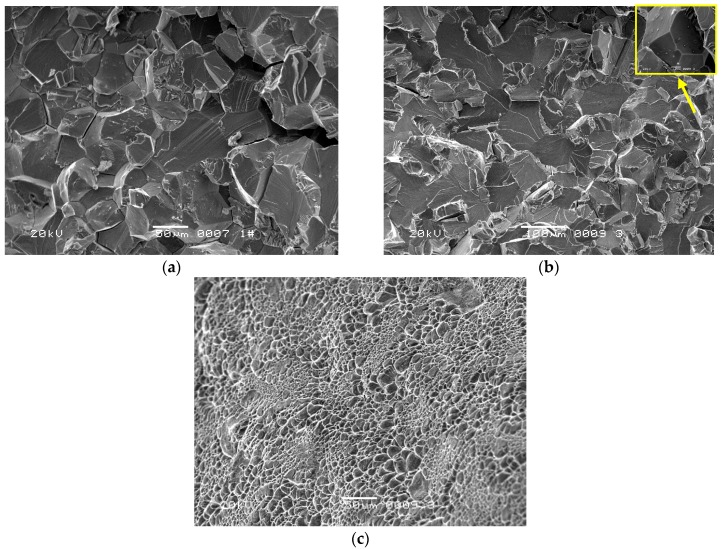
(**a**) Grain boundary cracking and cleavage morphology in Region I (50 μm); (**b**) Cleavage morphology and Grain boundary cracking in Region II (yellow square, 10 μm); (**c**) Dimple morphology in Region III (100 μm).

The #3 and #4 screws have the same microscopic appearance. Figure 5 shows low magnification of fracture surface the #4 screw. The fracture consists of three regions. Tiny cracks along grain boundaries can be seen in Region I (Figure 6a). Region II shows cleavage morphology, occupying an approximately half fraction of the fracture surface, with a small quantity of grain boundary cracking characteristics (yellow square in Figure 6b). Region III shows dimple morphology (Figure 6c).

### 2.3. Metallographic Observation

**Figure 7:**
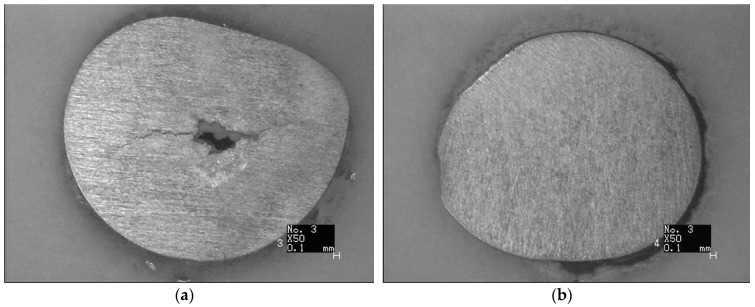
(**a**) Metallographic structure of axial through-wall crack; (**b**) Metallographic structure of #4 screw.

Metallographic specimens were cut perpendicular to the axial direction, 3 mm away from the fracture. Afterwards, the metallographic specimens were ground, polished, and etched (in a HF–HNO3 solution). The metallographic specimen of the #1 screw shows the axial crack similar to void (Figure 7a). In addition, no obvious cracks were observed for the #4 screw (Figure 7b). Furthermore, there is no evidence of material defects.

### 2.4. Chemical Constituents and Hydrogen Concentration

The chemical constituents of the failure screw were detected with an Oxford Instruments INCAx-Sight 6427 X-ray energy dispersive spectrometer (EDS). The beam size was set to 2 μm. Table 1 and Table 2 shows the chemical constituents of the #1 and #4 screw. The data of the two tables represent an average value. It can be concluded that the oxygen content on the defect and cleavage is high, while other regions meet the standard. Furthermore, there is no obvious impurity element.

The hydrogen concentration was measured via inert gas fusion analysis using a ONH836 apparatus (LECO Corporation, St. Joseph, MI, USA). The inert gas, purity helium, was used as the carrier gas during analysis. The hydrogen concentration test specimens, with a diameter of 3 mm and a gage length of 2 mm, were cut along the axial direction from the fractured screw. The hydrogen concentration of all the four fractured screws were below 100 ppm, which could meet the hydrogen content standards in titanium alloy manufacturing [17,18].

### 2.5. Stress Durability Embrittlement Testing

Although the result of hydrogen concentration shows that hydrogen content had met the standards, hydrogen embrittlement still cannot be ruled out completely. Hydrogen embrittlement of titanium is a common phenomenon. Its failure mechanism is known as delayed failure. Hydrogen may move into the areas of stress concentration and defective at low stresses by means of increasing diffusion. The delayed fracture of material with a high hydrogen content is related to plastic strains in the region of stress raisers and hydrogen segregations. Together with these internal stresses, external stresses promote further structural destabilization of the material with respect to hydrogen redistribution. Thus, under the loading condition, the structural instability of the material can be used as the criterion for judging the tendency towards delayed fracture [17].

Based on the delayed failure mechanism of hydrogen embrittlement, a stress durability embrittlement test (200 h proof testing for internal hydrogen embrittlement (IHE)) was designed to investigate whether the fracture of titanium alloy screws was caused by hydrogen embrittlement [19,20]. Six screws, the same batch of failure screws, were used as the test sample in the stress durability embrittlement test. The equation of the test tensile load is presented as follows:

*P* = σ_b_ × *S* × 75%, (1)

where σ_b_ is the tensile strength of the TB3 alloy, equal to 800 Mpa; *S* is the cross-sectional area, equal to 9.07 mm^2^; *P* is the test load. As a result, test load is equal to 5442 N, calculated via Equation (1). The screws were subjected to sustained tensile load at room temperature for 200 h, no fracture occurred.

### 2.6. Torsion Simulation Testing

**Figure 8:**
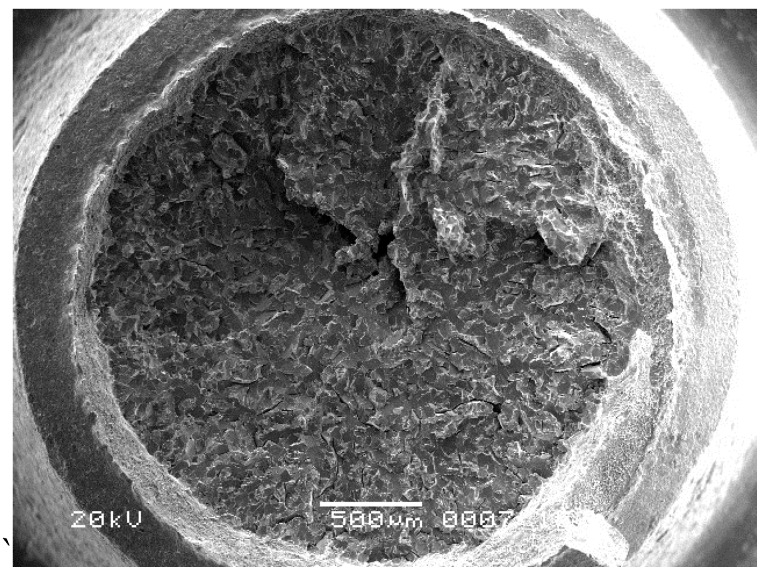
Low magnification of fracture surface in torsion simulation test (500 μm).

According to the principle of random sampling, fifty screws of the same production batch were employed to conduct the torsion simulation test. The torsion test was conducted using an MTS machine (CTT1501) at room temperature. The load increased 0.05 N·m every 0.2 s towards the maximum value of 2.5 N·m. The test condition was the same as the actual assembly process, and the torsion moment was no more than 2.5 N·m. During the test, when the torsion moment was not up to the specified value, two screws fractured. The fracture positions of the two screws were the same as that of the failure screws, which were located at the root of the screw thread. Fracture characteristics are the same as the failure ones, indicating that the failure is a batch problem, as shown in Figure 8.

## 3. Discussion

### 3.1. Fracture Mode Analysis

The screws fractured in the assembly process without cyclic stress and fatigue fracture characteristics, which illustrated that the failure was irrelevant to fatigue. In addition, creep ruptures at room temperature are serious issues for titanium alloys, but the fast assembly process does not give creep a chance to evolve. The screw fracture originated from the inside of the screws. Macro appearance shows that there is no obvious plastic deformation. The internal defects such as the axial crack and the tiny crack can be clearly seen in the center of the fracture surface. The fracture surface morphology of the screw around the crack is a cleavage river pattern. Through the above analysis, it can be concluded that the fracture mode is brittle.

### 3.2. The Mechanism for Brittle Fracture

Fracture caused by stress corrosions and liquid metal embrittlement starts at the surface, while the fractures in this batch of screws originated from the inside of the screws [21,22]. Furthermore, no corrosion products and impurity metal elements were found in the screws, so the brittle fracture of the screw is irrelevant to stress corrosion and liquid metal embrittlement.

The hydrogen concentration results and the proof test (survival for 200 h in the stress durability embrittlement test) led to the conclusion that the screws’ failures are validated as unaffected by hydrogen embrittlement [19,20].

**Figure 9:**
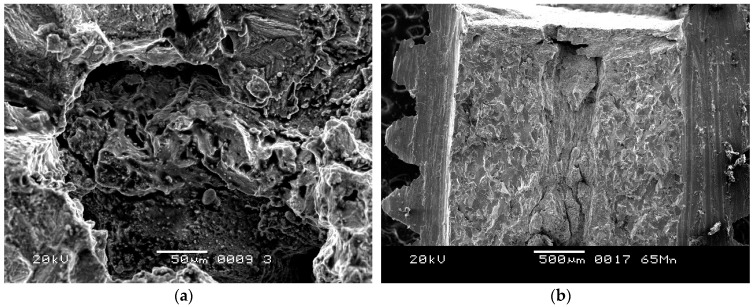
(**a**) Oxidation fracture morphology; (**b**) Morphology along the axial direction.

Due to the high stress concentration, screws usually fracture at the root of the thread. These fractures always start at the surface or sub-surface [23,24], whereas this batch of screws fractured at the root of the thread, originating from inside the screws. This is because of the internal defects, such as the axial crack and the tiny crack, in the screws, confirmed via microscopic and metallographic observations. During the assembly process, there is significant stress around the defects. When the stress exceeds the critical value, brittle fracture occurs. Figure 9 shows the oxidized screw, which is a failure in the production process of another batch, with significant defects. As is the typical oxidation morphology, small granular white objects can be seen in the fracture, as shown in Figure 9a. Figure 9b shows the morphology of the defects along the axial direction. Compared with Figure 9a, the small granular white objects can also be observed in Figure 4b, implying that the #1 screw was oxidized. However, the morphology of the oxidation was not observed in the fracture surface of the #4 screw. On the other hand, the EDS results, illustrated in Table 2, validated oxygen in the fracture surface of the #4 screw. This is due to the fact that oxygen dissolves in titanium to form an interstitial solid solution [25,26]. A titanium–oxygen solid solution and oxidation increase the brittleness of the titanium alloy [27]. Based on this analysis, we conclude that the main reason for brittle fracture of the screw is the internal defects, with oxygen increasing the brittleness.

### 3.3. The Causes of Internal Defects

The main cold working process of titanium alloy screws includes cold drawing, cold heading, and thread rolling. If the defects were induced in the cold working process, there should be surface defects, which is contrary what actually occurs. Thus, the internal defect of the screw is independent of the cold working process, proved by the high oxygen content around the defects, which cannot be induced in the cold working process. Therefore, these internal defects should be formed in the hot working process. The possible reasons for the formation of such defects in the hot working process include the following: (1) the material is porous; (2) the material is included; (3) the material is segregated; and (4) cracks are induced by hot forging.

If the material is porous and it is not healed by forging, the fracture morphology will be characterized by solidified morphology. However, the solidified characteristics were not obvious in the fracture surface of this batch screw. Besides, the result of the microscopic observation and the chemical constituents failed to show any evidence of inclusion, and segregation could have been responsible for the defects.

In this paper, the grain boundary cracking characteristics were observed around the internal defects, such as the axial crack and the tiny crack in the middle of the fracture surface. Thus, the internal defects of screws should result from grain boundary cracking caused by hot forging. After forming the defects, it is further extended in the subsequent cold working process and consistent with the axial direction.

## 4. Conclusions

(1) The fracture mode of the screws is brittle. The main reason for the brittle fracture is the internal defects, around which oxygen content is high, increasing brittleness.
(2) The axial crack can be clearly seen in the center of the #1 screw fracture surface. Grain boundary cracking characteristics were observed around the axial crack.
(3) Tiny cracks and cleavage morphology with grain boundary cracking characteristics is the major fracture surface morphology of the #4 screw.
(4) The hydrogen concentration and stress durability embrittlement test results demonstrate that the screws’ failures are validated as unaffected by hydrogen embrittlement.
(5) The internal defects resulted from grain boundary cracking caused by hot forging. After the formation of the defects, it was further extended in the subsequent cold working process and consistent with the axial direction.

## Figures and Tables

**Table 1:** Chemical constituents of the #1 screw (wt %).

Region	Al	Fe	V	Mo	O	Ti
cleavage	3.42	0.66	6.52	6.1	5.5	77.84
defect	3.3	0.77	8.06	5.8	8.91	73.16
margin of the fracture surface	3.53	0.87	7.51	9.60	–	78.49
standard	2.7–3.7	0.8–1.2	7.5–8.5	9.5–11.0	–	Balance

**Table 2:** Chemical constituents of the#4 screw (wt %).

Region	Al	Fe	V	Mo	O	Ti
I	3.35	0.73	8.07	5.6	9.11	73.14
II	3.49	0.59	5.63	5.29	6.75	77.96
III	3.1	0.81	7.9	10.3	–	77.89
standard	2.7–3.7	0.8–1.2	7.5–8.5	9.5–11.0	–	Balance

## Abbreviations

The following abbreviations are used in this manuscript:

TC4	Ti-6Al-4V
BT16	Ti–3Al–4.5V–5Mo
TB3	Ti-10Mo-8V-1Fe-3.5Al
SEM	scanning electron microscopy
EDS	energy dispersive spectrometer
IHE	internal hydrogen embrittlement
